# Safety and outcome of definitive chemoradiotherapy in elderly patients with oesophageal cancer

**DOI:** 10.1038/sj.bjc.6604749

**Published:** 2008-10-28

**Authors:** D Tougeron, F Di Fiore, S Thureau, N Berbera, I Iwanicki-Caron, H Hamidou, B Paillot, P Michel

**Affiliations:** 1 Digestive Oncology Unit, Department of Gastroenterology, Rouen University Hospital, Northwest Canceropole, 1 rue de Germont, Rouen Cedex 76031, France; 2 Department of Radiotherapy, CRLCC Becquerel, Northwest Canceropole, rue d’Amiens, Rouen 76000, France

**Keywords:** oesophageal cancer, elderly patients, definitive chemoradiotherapy

## Abstract

Little is known about chemoradiotherapy (CRT) in elderly patients with a locally advanced oesophageal cancer (OC). The aim of our study was to evaluate the tolerance and the outcome of elderly patients older than 70 years treated with CRT for a non-metastatic OC. Chemoradiotherapy was based on radiotherapy combined with a cisplatin-based chemotherapy. Clinical complete response (CCR) to CRT was evaluated on upper digestive endoscopy and computed tomography scan 6–8 weeks after CRT completion. One hundred and nine consecutive patients were included. A CCR was observed in 63 patients (57.8%) and 2-year survival was 35.5%. Adverse events ⩾grade 3 were observed in 26 (23.8%) patients. Chemotherapy dose reduction, chemotherapy delays more than 1 week, and treatment discontinuation were observed in 33 (30.3%), 45 (41.3%), and 17 patients (15.6%), respectively. Comorbidity index according to Charlson score was significantly associated with treatment tolerance. In multivariate analysis, a CCR to CRT (*P*<0.01), a dose of radiotherapy ⩾80% (*P*=0.02), and a Charlson score ⩽2 (*P*=0.046) were identified as independent prognostic factors of overall survival. These results suggest that CRT could be considered as an effective treatment without major toxicity in elderly patients with OC.

Management of elderly patients with cancer is a therapeutic challenge. It is well established that cancer occurs primarily in elderly patients, with approximately two-thirds of cancer-related death in the elderly over 60 years and 25% in patients older than 80 years. Oesophageal cancer (OC) is the eighth most common cancer and the sixth cause of cancer mortality worldwide (Parkin, 2001). In France, approximately 54% of OC occurs in patients older than 65 years, with 23% over 75 years (Remontet *et al*, 2003).

The most relevant treatment modalities in elderly patients with OC remain a subject of debate. Although survival improvement has been observed during the past decade, prognosis of OC has remained significantly influenced by age (Bouvier *et al*, 2005; Cranea *et al*, 2007). A recent population-based study including patients with locoregional OC showed a difference in treatment and survival according to age, comorbidity, race, and geographical region (Steyerberg *et al*, 2007). It has also been reported that elderly patients were less likely to undergo surgery and chemotherapy, which was however partially explained by their comorbidity (Steyerberg *et al*, 2007; Ruol *et al*, 2007a). Currently, definitive chemoradiotherapy (CRT) based on the 5-fluorouracil–cisplatin (5FU–CDDP) regimen has been considered in curative intent in locally advanced or inoperable non-metastatic OC (Kleinberg and Forastiere, 2007; National Cancer Institute recommendations, 2008; Stahl and Oliveira, 2008).

Little is known about the efficacy of CRT in elderly patients with a locally advanced OC. Indeed, data on tolerance and outcome in these patients remain lacking. In the two recent randomised trials that investigated the efficacy of CRT, the mean ages of included patients were 59.1 and 57 years, respectively (Bedenne *et al*, 2007; Stahl *et al*, 2005). Moreover, patients up to 70 years were not eligible in the Stahl *et al* (2005) trial. More recently, in a limited number of 25 patients older than 65 years, Anderson *et al* (2007) reported that definitive chemoradiation using two cycles of 5FU plus mitomycin-C associated with 50.4 Gy radiation could be considered as an active regimen with moderate toxicity.

The aim of our study was to evaluate the safety and the efficacy of CRT in elderly patients older than 70 years treated for a non-metastatic OC.

## Patients and methods

### Patient's inclusion

All consecutive patients older than 70 years with a non-metastatic OC treated with definitive CRT in Digestive Oncology Unit of Rouen University Hospital between January 1994 and June 2007 were included. The ethical committee approved the procedure and, due to the retrospective analysis with majority of died patients, any patient contentment was necessary. Patient's baseline characteristics (dysphagia, WHO performance status, weight loss, albumine rate, nutritional intervention namely enteral nutrition, and/or endoscopic dilation) were collected. Degree of dysphagia was evaluated using the Atkinson (1977) score.

All patients had a histologically proven OC without visceral metastasis at the time of diagnosis and were treated with definitive CRT (Herskovic *et al*, 1992; Stahl *et al*, 2005; Bedenne *et al*, 2007). Patients who had a cancer of the lower and the upper oesophagus associated with an involvement of the coeliac or the sus-clavicular lymph node areas (M1a stage), respectively, were also selected.

We used the Charlson score, which is widely used in comorbidity index, for the analysis of patient's comorbidities. The Charlson score consists of 19 different disease comorbidity categories (coronary artery disease, congestive heart failure, chronic pulmonary disease, peptic ulcer disease, peripheral vascular disease, liver disease, cerebrovascular disease, connective tissue disease, diabetes, dementia, renal disease, prior tumour, and AIDS), each allocated a weight of 1–6 based on the adjusted relative risk of 1-year mortality and summed to provide a total score.

### Tumour staging

The tumour staging was based on the 1983 AJCC staging system according to published recommendations (Coia *et al*, 2000). Tumour evaluation was based on oesophagoscopy, barium oesophagography, chest and abdominal computed tomography (CT scan) (Bosset *et al*, 1997), and oesophageal ultrasonography (Tio *et al*, 1990) when it was feasible. Tumour baseline characteristics (TNM stage, location, length, diameter, and histologic type) were collected. The tumour length was defined by oesophagoscopy±barium oesophagography and tumour diameter by endoscopic ultrasound and/or CT scan.

### Treatment and CRT regimen

Patients received a CRT regimen based on the CDDP and 5FU chemotherapy combination described by Herskovic *et al* (1992) or on the CDDP/irinotecan chemotherapy combination described by Michel *et al* (2006). The Herskovic CRT regimen was based on four CDDP/5FU chemotherapy courses, which were delivered concomitantly with 50–55 Gy radiotherapy (weeks 1–5). Starting dose regimens were 1000 mg m^−2^ at days 1–5 for 5FU and 75 mg m^−2^ at day 1 for CDDP. The CRT regimen based on the CDDP/irinotecan chemotherapy combination has been recently reported in a phase II multicentric trial and consisted of eight chemotherapy courses delivered concomitantly with 50–55 Gy radiotherapy (courses five to eight). Starting dose regimens were 60 mg m^−2^ for irinotecan and 30 mg m^−2^ for CDDP at each cycle. Radiotherapy was delivered 5 days per week at 1.8 or 2 Gy day^−1^ in both CRT regimens. The target volume of radiotherapy was the macroscopic tumour and enlarged lymph nodes, if any, surrounded by 5-cm proximal and distal margins and a 2-cm radial margin. The target was extended to the inferior cervical area in cases of tumours located above the carina. The specified dose was delivered at the intersection of the central axis of the beams, according to international guidelines.

Twenty-five patients have an initial dose reduction due to their age or comorbidities. Among these patients, the chemotherapy start dose was generally 50 or 75% of complete dose.

At day 1 of each chemotherapy course, toxicities related to the treatment were evaluated using the National Cancer Institute Common Toxicity Criteria (NCI-CTC, version 2.0). We also noted a delay of chemotherapy and CRT stop for toxicity. Percentage of planned radiotherapy and chemotherapy dose was calculated.

### Response to CRT and follow-up

Patients were considered to have a clinical complete response (CCR) to CRT when no residual tumour was identified on upper digestive endoscopy and when no metastatic disease occurrence was observed on CT scan. This evaluation was performed 6–8 weeks after CRT completion. The follow-up was performed on a clinical basis, with upper digestive endoscopy with biopsy and chest and abdominal CT scans every 3 months. Local recurrence was defined by positive biopsy at upper digestive endoscopy. Salvage surgery in patients without CCR or with local recurrence and absence of metastases were also collected. Follow-up data were updated in December 2007. Among patients alive at 6 months, median follow-up was 20.5 months (6–127 months).

### Statistical analysis

Overall survival was calculated from the date of CRT initiation until the date of death or the date of last follow-up. Survival curve was established using Kaplan–Meier method. Disease-free survival was estimated from the date of the first day of CRT initiation to the time of documented failure (local recurrence or metastasis occurrence) or the date of the last follow-up for those remaining with CCR. Specific survival excluded patients who died of other causes than cancer or treatment. Predictive factors of CCR to CRT were determined by a univariate analysis and further evaluated in multivariate logistic regression analysis. Predictive factors of survival were studied by a univariate analysis and further evaluated in multivariate Cox regression analysis to estimate the hazard ratio (HR) with 95% confidence interval (CI). Nine pre-defined baseline variables for the univariate analysis were sex, age, WHO performance status <2, initial weight loss <10%, albumine ⩾30 g l^−1^, Charlson score ⩽2, dose of cisplatin ⩾80%, dose of radiotherapy ⩾80%, and CCR to CRT. Any variables reaching *P*=0.05 were introduced in multivariate analysis. We also performed an analysis with stratification for age ⩾75 years *vs* <75 years and patients with or without comorditidies (Charlson index ⩽2 *vs* >2). All statistical analyses were performed with a two-side significance value of 0.05. Statistical analysis was performed using the Statview software (Statview for Windows, SAS Institut Inc., version 5.0).

## Results

### Patient and tumour characteristics

One hundred and nine patients over 70 years were analysed. Clinical baseline characteristics are detailed in Table 1. Mean age was 74.4±3.7, ranging from 70 to 88 years. There were 38 patients (34.9%) aged more than 75 years. Majority of patients (79.8%) had a good WHO performance index (0 or 1). Majority of patients had a severe dysphagia ⩾2 (70.6%) and 33.0% had an initial weight lost ⩾10%.

There were mainly T3 stage tumours (*n*=76, 69.7%) and squamous cell carcinoma (*n*=77, 70.6%) (Table 2). Majority of tumours were more than 5 cm in length (55.0%), and 29.3% had a diameter above 3 cm.

Median Charlson score was 1 (range 0–6) (available in 88 patients). Twenty-seven patients (30.7%) had Charlson score ⩾2 and 84% Charlson score ⩾1. Majority of patients were autonomous at home (96.6%). Patients took, on an average, three different medications per day. The prevalence of patients with comorbidities was 84%. Thirteen patients (14.8%) had chronic obstructive pulmonary disease, 12 patients (13.6%) had a prior or concurrent malignancy, 11 patients (12.5%) had diabetes, and 10 patients (11.4%) had peripheral vascular disease.

### Treatment regimen and tolerance

Eleven patients (10.1%) were started on enteral nutrition and 17 (15.6%) on endoscopic dilatation before starting the treatment (Table 3). Ninety-eight patients (89.9%) had treatment with 5FU/CDDP. Sixty-three (57.8%) patients received ⩾80% of cisplatin planned dose and 85 (78%) received ⩾80% of radiotherapy planned dose. Sixty-two patients (56.9%) experienced adverse effects ⩾grade 2, mainly vomiting and neutropaenia. Twenty-four patients experienced grade 3 toxicity (22.0%), four an adverse effect grade 4, with one toxic death (neutropaenia with peritonitis caused by diverticular sigmoiditis). These adverse events conducted to a treatment-related hospitalisation in 18 patients (16.5%). Dose reduction for toxicity was performed in 33 patients (30.3%), a chemotherapy delay of more than 1 week was required in 45 patients (41.3%), and CRT discontinuation was necessary in 17 patients (15.6%). Finally, 42 patients (38.5%) completed the planned CRT regimen.

### Response to CRT, survival and outcome

**Figure 1:**
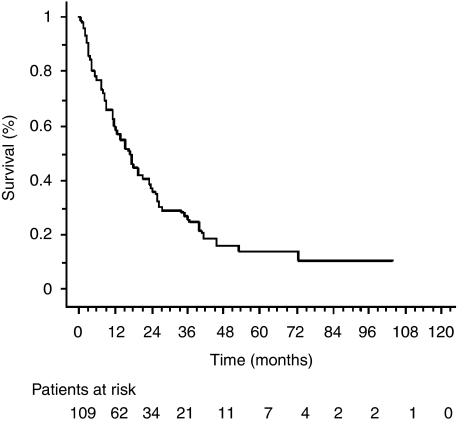
Overall survival. The median overall survival was 15.2±2.8 months.

Median overall survival was 15.2±2.8 months and disease-free survival was 8.3±7.3 months (Figure 1) (Table 4). Two-year and 5-year survival rates were 35.5% (95% CI: 30.8–40.2) and 12.8% (95% CI: 9.2–16.4), respectively. Distant metastasis occurred in 31 patients (28.4%) during follow-up. Cancer was the cause of death in 73 patients (80.2%) among the patients who died (*n*=91).

**Figure 2:**
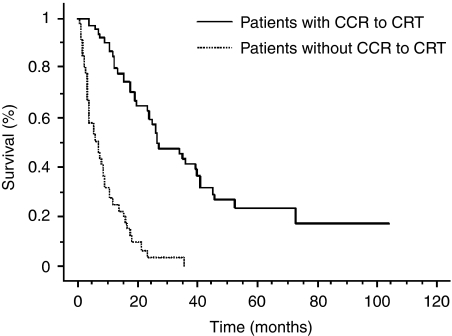
Overall survival in responders and non-responders to CRT. The median overall survival was 27.2±5.1 months in responder patients as compared with 6.0±2.5 months in non-responder patients (*P*<0.01). CCR=clinical complete response; CRT=chemoradiotherapy.

A CCR to CRT was observed in 63 patients (57.8%), and 26 (23.8%) of them had no recurrence at follow-up (median follow-up was 20.5 months and eight patients were lost to follow-up). Median overall survival in patients with CCR was 27.2±5.1 months as compared with 6.0±2.5 months in non-responders (*P*<0.01) (Figure 2). Among responders to CRT, 21 patients (33.3%) had a local recurrence, with a median time of 13.4±2.2 months. Two patients with local recurrence underwent surgery but the majority of patients were treated by oesophageal stent (*n*=9), chemotherapy (*n*=4), or the best supportive care (*n*=6). In non-responder patients to CRT, a salvage surgery was performed in five patients. One patient died after surgery and three had local or metastasis recurrence. Patients without CCR to CRT and surgical contraindications were treated by oesophageal stent (*n*=7), chemotherapy (*n*=3), or the best supportive care.

### Prognostic factors of CCR to CRT and overall survival

Predictive factors of a CCR to CRT in univariate analysis were a WHO performance status <2 (*P*=0.02), an initial weight loss <10% (*P*=0.01), a dose of cisplatin ⩾80% (*P*<0.01), and a dose of radiotherapy ⩾80% (*P*<0.01) (Table 5). In multivariate analysis, adjusted for sex and age, a dose of radiotherapy ⩾80% (HR=12.1, 95% CI: 3.0–49.4; *P*<0.01) and a dose of cisplatin ⩾80% (HR=3.4, 95% CI: 1.3–9.1; *P*=0.01) were identified as independent prognostic factors of CCR.

Predictive factors of overall survival in univariate analysis were a CCR to CRT (*P*<0.01), a WHO performance status <2 (*P*<0.01), an initial weight loss <10% (*P*=0.03), a dose of cisplatin ⩾80% (*P*=0.03), a dose of radiotherapy ⩾80% (*P*<0.01), and a Charlson score ⩽2 (*P*=0.02). T stage and age < or >75 years were not predictive factors of overall survival in univariate analysis (T stage, *P*=0.85 and age < or >75 years, *P*=0.15). In multivariate analysis, adjusted for sex and age, a CCR to CRT (27.2±5.1 months in responders *vs* 6.0±2.5 months in non-responders; HR=4.9, 95% CI: 2.5–9.5; *P*<0.01), a dose of radiotherapy ⩾80% (21.2±7.0 months if dose of radiotherapy ⩾80% *vs* 3.3±1.0 months if dose of radiotherapy <80%; HR=2.3, 95% CI: 1.3–4.2; *P*=0.02), and a Charlson score ⩽2 (13.9±3.6 months if Charlson score ⩽2 *vs* 4.1±2.6 months if Charlson score >2; HR=2.1, 95% CI: 1.0–4.5; *P*=0.046) were identified as independent prognostic factors of overall survival.

### Factors that influence treatment tolerance

Age ⩾75 years was associated with worse creatinine clearance (*P*<0.01) and more chemotherapy dose reduction at treatment onset due to age (*P*<0.01), but this had no influence on total CRT dose, adverse events, CCR to RCT, or overall survival. Patient with a dose reduction at treatment onset was older but age did not influence chemotherapy dose. This result was probably explained by the fact that the patient with a dose reduction up front has a secondary increase of chemotherapy dose if the tolerance was good (nine patients).

Charlson score ⩽2 *vs* >2 did not influence adverse events, chemotherapy delay, CRT dose, or CCR to CRT, but only overall survival. Nevertheless, patients with Charlson score ⩾1 experienced more adverse events grade ⩾2 (76.5 *vs* 51.2%; *P*=0.01) and chemotherapy delay (66.7 *vs* 39.5%; *P*=0.02).

## Discussion

This study suggested that definitive CRT in elderly patients with an OC was an effective treatment without a major increase in adverse events. Moreover, our results showed that CRT in elderly patients produced a similar response rate and overall survival as usually reported in younger patients treated with the same regimen. These findings suggest that a treatment based on a CRT regimen may be discussed in elderly patients. Improvements in general health care and increased life expectancy have resulted in more elderly patients with an OC. At 75 years of age, life expectancy was more than 10 years (Arias, 2006). Therefore, if there are no major comorbidities, elderly patients with OC should benefit from curative treatment.

Definitive CRT is considered as a feasible non-surgical treatment for patients with a locally advanced OC with approximately 50–65% CCR rate, 17–26 months of median overall survival, and 30–40% 2-year survival rate (Minsky *et al*, 1999; Ohtsu *et al*, 1999; Coia *et al*, 2000; Seitz *et al*, 2000; Stahl *et al*, 2005; Michel *et al*, 2006; Di Fiore *et al*, 2006a; Bedenne *et al*, 2007). Interestingly, our results in elderly patients were relatively close to those reported in these series, with a CCR to CRT of 57.8% and a 2-year overall survival rate of 35.5%. However, overall median survival in our study (15.2 months) was slightly lower as compared with that reported in previous randomised trials. These results were strictly similar to those published by our group on series including non-selected patients with an oesophageal carcinoma treated with the same CRT regimens (Michel *et al*, 2006; Di fiore *et al*, 2006a, 2006b; Tougeron *et al*, 2008). Indeed, the mean age of patients ranged from 39 to 88 years in these series and we found that the complete response to CRT was 50–80% and the overall survival was 16–20 months as in this study in elderly patients. Although age and comorbidity were associated with higher difficulties encountered during treatment, no significant association was found between these factors and survival in our study. Moreover, the difference in median overall survival could be partially explained by a selection bias in these prospective trials, whereas our study possibly reflected the outcome of non-selected patients. In a recent study, predictive factors of prognosis in locally advanced OC from the randomised FFCD 9102 trial in the multivariate analysis were age more than 65 years, inability to ingest solid food, and the presence of more than three neoplastic coeliac lymph nodes on endoscopic ultrasonography (Burtin *et al*, 2008).

Anderson *et al* (2007) reported significant results from a single institution experience of chemoradiation in 25 elderly patients older than 65 years with OC. On the basis of a median follow-up of 32 months, the CCR rate was 68% and the 2-year survival rate was 64% (95% CI: 45–83) in this study. The overall survival was very good probably because there was a careful selection of patients using Charlson score and data were only available on 25 patients with a very significant CI for survival rate. Different findings were found in the Takeuchi *et al* (2007) study in which results of CRT based on 5FU/CCDP and 60 Gy of radiation were compared between 33 elderly patients *vs* 145 non-elderly. In fact, a significant worse survival and higher CRT discontinuation and toxicity were reported in elderly. These contradictory results strongly support that more data on larger population, as in our study, are required to accurately estimate the safety and efficacy of the CRT approach in an elderly population. Local recurrence and metastasis occurrence in our study were in accordance with randomised trials (Minsky *et al*, 1999; Seitz *et al*, 2000; Bedenne *et al*, 2007).

Salvage oesophagectomy has been reported in a small group of elderly patients who did not respond to CRT. Five patients underwent surgery in our series and only one in the study of Anderson *et al* (2007). Death due to post-operative complications occurred in one patient in our study and also in one patient in the Anderson *et al* (2007) study. Although a significant improvement has been obtained in post-operative resuscitation, oesophagectomy for OC remains associated with significant morbidity and mortality rates. In this context, surgical approach in patients older than 70 years with OC is still debated because of potentially higher post-operative complication rates (Poon *et al*, 1998; Law *et al*, 2004; Moskovitz *et al*, 2006). On the basis of a population of 421 patients with intrathoracic squamous cell carcinoma treated with surgical resection, Law *et al* (2004) found that age was significantly associated with pulmonary complications and hospital mortality. In a single institution study on 751 patients including 31 patients older than 80 years, Moskovitz *et al* (2006) reported that post-operative death and hospital length of stay were significantly worse in elderly patients, independently of comorbidity. In contrast, Ruol *et al* (2007b, 2007c) recently showed similar short- and long-term post-operative outcomes in elderly patients as compared with younger patients. This difference could be due to difference in surgical and resuscitation experience between centres. Median survival after oesophagectomy remains poor in elderly patients, ranging from 6 to 27 months, with major post-operative mortality ranging from 4.7 to 7.2% (Thomas *et al*, 1996; Poon *et al*, 1998; Sabel *et al*, 2002; Internullo *et al*, 2008).

In our study, the planned treatment was achieved in only 38.5% of patients, and 53.2% of them required dose adjustment. However, grade 4 toxicity was observed only in three patients (2.7%), with two treatment-related deaths (1.8%). In the Anderson *et al* (2007) study, 88% of patients completed the planned CRT, grade 4 toxicity occurred in 16%, and no treatment-related deaths were observed. Moreover, severe adverse events (grade 3 or 4) were observed in 23.8% of patients in this study and 31% of patients randomised in the definitive CRT arm of FFCD 9102 study. Chemoradiotherapy tolerance in ‘selected’ elderly patients with an OC was acceptable as compared with younger patients, but a dose reduction was frequently necessary due to adverse events. Age ⩾75 years had no influence on adverse events. These results underlined that age criteria alone is not sufficient for guidance of therapy and that better characterisation of patients with Charslon score, for example, may be helpful for decision making. 5-Fluorouracil tolerance was the same in the elderly and younger patients (Popescu *et al*, 1999). Moreover, a recent study on patients with an advanced oesophagogastric adenocarcinoma suggested that cisplatin-based chemotherapy toxicities did not increase with age (Trumper *et al*, 2006). In patients with other cancers (rectum, lung, or head and neck cancers) treated with concomitant CRT, the same results were observed. In these elderly patients treated with CRT for other tumours, no major toxicity was observed with similar survival rates than in younger patients (Airoldi *et al*, 2004; Pasetto *et al*, 2006; Semrau *et al*, 2007).

The main reported predictive factors of response to CRT and overall survival were WHO performance status, nutritional status, treatment dose, and TNM stage (Coia *et al*, 2000; Polee *et al*, 2003; Rohatgi *et al*, 2005; Di Fiore *et al*, 2006a). No major difference was found, in our multivariate analysis, in predictive factors of CCR to CRT and survival in elderly patients. Although age and Charlson score were associated with nutritional impairment and a greater chemotherapy dose reduction, our analysis also underlined that the success rate of CRT in OC was not dependant on age.

In conclusion, our results showed that definitive CRT could be considered as an effective treatment with no significant toxicity in elderly patients with OC.

## Conflict of interest

The authors confirm that there is no conflict of interest.

## Figures and Tables

**Table 1:** Patient characteristics

	***n*=109**
Age (s.d., min–max)	74.4±3.7 (70–88)
Sex ratio (men/women)	90/19
	
*WHO performance status* (n, %)	
0	24 (22.0)
1	63 (57.8)
2	22 (20.2)
WHO <2	87 (79.8)
	
*Atkinson dysphagia score* (n, %)	
0	5 (4.6)
1	27 (24.8)
2	55 (50.4)
3	15 (13.8)
4	7 (6.4)
Dysphagia stage ⩾2	77 (70.6)
	
Initial BMI (kg m^−2^, s.d.)	24.9±6.8
Initial weight loss (%, s.d.)	7.7±6.6
Initial weight loss ⩾10% (*n*, %)	36 (33.0)
Initial albumin (g l^−1^, s.d.)	37.7±5.1
	
Creatinine clearance (ml min^−1^, s.d.)	73.2±22.3
	
Charlson score^a^	
Median (min–max)	1 (0–6)
Charlson score ⩾2	27 (30.7%)

BMI=body mass index; *n*=number of patients; s.d.=standard deviation.

a Available for 88 patients.

**Table 2:** Tumour characteristics

	***n*=109**
*TNM stage* (n, %)	
T1N0	2 (1.8)
T1N1	0
T2N0	13 (11.9)
T2N1	5 (4.6)
T3N0	32 (29.3)
T3N1	44 (40.4)
T4N0	0
T4N1	2 (1.8)
Coeliac lymph nodes M1a	6 (5.5)
Sus-clavicular lymph nodes M1a	0
Unknown (M0)	5 (4.6)
	
*Tumoral stage* (n, %)	
Stage I	2 (1.8)
Stage II	50 (45.9)
Stage III	46 (42.2)
Stage IV (M1a)	6 (5.5)
Unknown (M0)	5 (4.6)
	
*Oesophagus tumoral location* (n, %)	
Lower one-third	52 (47.7)
Middle one-third	36 (33.3)
Upper one-third	21 (19.3)
	
Mean tumour length (cm, s.d.)	5.2±2.1
Mean tumour length ⩾5 cm (n, %)	60 (55.0)
Mean tumour diameter (cm, s.d.)	2.7±1.2
Mean tumour diameter ⩾3 cm (*n*, %)	32 (29.3)
	
*Histological type (n*, %)	
Squamous cell carcinoma	77 (70.6)
Adenocarcinoma	28 (25.7)
Indifferentiated	4 (3.7)
	
*Histological differentiation*(n, %)	
Well differentiated	26 (23.8)
Fairly differentiated	22 (20.2)
Poorly differentiated	12 (11.0)
Indifferentiated	5 (4.6)
Unknown	44 (40.4)
	
CT scan (*n*, %)	107 (98.2)
Echoendoscopy (*n*, %)	20 (18.3)

*n*=number of patients; s.d.=standard deviation.

**Table 3:** Treatment regimen and toxicity

	***n*=109**
*Treatment before CRT initiation (n*, %)	
Enteral nutrition by nasogastric tube	7 (6.4)
Enteral nutrition by stomy	4 (3.7)
Endoscopic dilation	17 (15.6)
Oesophageal stent	2 (1.8)
	
*Chemotherapy regimen* (n, %)	
Irinotecan/CDDP	10 (9.2)
5-FU/CDDP	98 (89.9)
Other	1 (0.9)
	
*Mean chemotherapy course*	
Irinotecan/CDDP regimen	6.2±2.6
5-FU/CDDP regimen	3.6±1.8
	
Mean radiotherapy dose (grays)	49.0±13.9
	
*Treatment dose*	
% of cisplatin planned dose (%, s.d.)	69.0±27.4
% of cisplatin planned dose ⩾80%	63 (57.8)
% of radiotherapy planned dose (%, s.d.)	89.8±22.9
% of radiotherapy planned dose ⩾80%	85 (78.0)
	
Patients with adverse effects ⩾grade 2 (*n*, %)	62 (56.9)
Patients with treatment delay more than 1 week (*n*, %)	45 (41.3)
Patients with treatment discontinuation (*n*, %)	17 (15.6)
	
Patients with chemotherapy dose reduction (*n*, %)	58 (53.2)
Due to adverse events	33 (30.3)
Due to age	25 (22.9)
	
*Patients with treatment toxicity ⩾grade 2* (n, %)	
Neutropaenia	27 (24.8)
Vomiting	16 (14.7)
Mucitis	15 (13.8)
Infection	13 (11.9)
Diarrhoea	8 (7.3)
Renal insufficiency^a^	6 (5.5)

CDDP/irinotecan regimen=cisplatin and irinotecan chemotherapy combination; CRT=chemoradiotherapy; *n*=number of patients; s.d.=standard deviation; 5-FU/CDDP regimen=cisplatin and 5-fluorouracil chemotherapy combination.

a Diminution of creatinine clearance under 50 ml min^−1^ after starting of chemotherapy.

**Table 4:** Patients’ outcome and survival

		***n*=109**
*Outcome in all patients*		*n*=109
CCR to CRT (*n*, %)		63 (57.8)
Overall survival (months, s.d.)		15.2±2.8
Specific survival		19.5±1.9
Disease-free survival (months, s.d.)		8.3±7.3
Metastasis occurrence (*n*, %)		31 (28.4)
Mean time of metastasis occurrence (months, s.d.)		15.4±3.9
		
*Outcome in patients with CCR*		*n*=63
Local recurrence (*n*, %)		21 (33.3)
Mean time to local recurrence (months, s.d.)		13.4±2.2
Metastasis occurrence (*n*, %)		21 (33.3)
Mean time to metastasis occurrence (months, s.d.)		17.4±4.7
Median survival (months, s.d.)		27.2±5.1
Patient without recurrence		26 (23.8%)
		
*Outcome in non-responders to CRT*		*n*=46
Median survival (months, s.d.)		6.0±2.5
		
*Causes of death*		*n*=91
Cancer		73 (80.2%)
Treatment		2 (2.2%)
Others		14 (15.4%)

CCR=clinical complete response; CRT=chemoradiotherapy; *n*=number of patients; s.d.=standard deviation.

**Table 5:** Univariate and multivariate analysis of CCR to CRT and overall survival

	**Univariate analysis**		**Multivariate analysis**	
	**Response rate or median survival**	***P*-value**	**HR (95% CI)**	***P*-value**
*Predictive factors of CCR to CRT* a				
WHO performance status <2		*P*=0.02		NS
Initial weight loss <10%		*P*=0.01		NS
Albumine ⩾30 g l^−1^		*P*=0.61		
Dose of cisplatin ⩾80%		*P*<0.01	12.1 (3.0–49.4)	*P*<0.01
Yes	76.1%			
No	44.4%			
Dose of radiotherapy ⩾80%		*P*<0.01	3.4 (1.3–9.1)	*P*<0.01
Yes	70.6%			
No	12.5%			
Charlson score ⩽2		*P*=0.76		
				
*Predictive factors of overall survival* b				
CCR to CRT		*P*<0.01	4.9 (2.5–9.5)	*P*<0.01
Yes	27.2±5.1			
No	6.0±2.5			
WHO performance status <2		*P*<0.01		NS
Initial weight loss <10%		*P*=0.03		NS
Albumine ⩾30 g l^−1^		*P*=0.43		
Dose of cisplatin chemotherapy ⩾80%		*P*=0.03		NS
*Dose of radiotherapy ⩾80*%		*P*<0.01	2.3 (1.3-4.2)	*P*=0.02
Yes	21.2±7.0			
No	3.3±1.0			
*Charlson score ⩽2*		*P*=0.02	2.1 (1.0–4.5)	*P*=0.046
Yes	13.9±3.6			
No	4.1±2.6			

CCR=clinical complete response; CI=confidence interval; CRT=chemoradiotherapy; HR=hazard ratio; NS, nonsignificant.

a Multivariate logistic regression analysis adjusted on sex and age.

b Multivariate Cox regression analysis adjusted on sex and age.
